# Distal Bypass Improves Skin Perfusion Pressure at the Whole Foot Regardless of Angiosomes in Patients with Chronic Limb-Threatening Ischemia

**DOI:** 10.3400/avd.oa.23-00105

**Published:** 2024-04-10

**Authors:** Fukashi Serizawa, Yoshiyuki Nakano, Munetaka Hashimoto, Yoshihisa Tamate, Hiroko Sato, Masato Ohara, Keiichiro Kawamura, Daijiro Akamatsu, Takashi Kamei

**Affiliations:** 1 Division of Vascular Surgery, Department of Surgery, Tohoku University Graduate School of Medicine, Sendai, Miyagi, Japan; 2 Division of Vascular Surgery, Department of Surgery, Ishinomaki Red Cross Hospital, Ishinomaki, Miyagi, Japan; 3 Division of Vascular Surgery, Department of Surgery, Japan Community Health Care Organization Sendai Hospital, Sendai, Miyagi, Japan; 4 Division of Vascular Surgery, Department of Surgery, Iwate Prefectural Isawa Hospital, Osyu, Iwate, Japan

**Keywords:** skin perfusion pressure, distal bypass, angiosome

## Abstract

**Objectives:** Distal bypass surgery’s effect on tissue blood pressure beyond a focal angiosome remains debated. This study assessed tissue blood pressure in both direct revascularized angiosome (DRA) and indirect revascularized angiosome (IRA) after bypass surgery, utilizing repeated skin perfusion pressure (SPP) measurements.

**Methods:** Twenty-nine limbs in 27 chronic limb-threatening ischemia (CLTI) patients (22 males and five females, age: 70.2 ± 9.3 years) who received distal bypass surgery were enrolled. SPP measurements were conducted for the DRA and IRA at 10 time intervals, encompassing both preoperative and postoperative periods of every 3–5 days until 30 days.

**Results:** In total, 486 SPP measurements were collected from 58 measurement sites, and the transition of the SPP at the DRA was 35.4–62.5–59.5–70.2–58.2–62.2–63.1–63.6–63.8–73.4 mmHg and IRA was 29.4–53.4–53.7–58.8–51.3–63.1–47.9–62.1–57.6–61.0 mmHg. No significant differences were observed between SPP at the DRA and IRA. Fifteen wounds on the DRA (63%) and five on the IRA (100%) healed.

**Conclusion:** Distal bypass improves SPP in both direct and IRAs of CLTI patients. These data indicated distal bypass improves tissue blood flow at entire foot regardless of angiosomes.

## Introduction

Peripheral artery disease is common among the elderly, with 15%–20% of people over 70 years are affected.^1^^,^^2)^ Up to 10% of peripheral artery disease cases progress to chronic limb-threatening ischemia (CLTI),^3)^ whose annual incidence is estimated to be 500–1000 per million individuals.^4)^ Patients with CLTI often have tissue loss, and appropriate tissue blood flow with revascularisation is required. Skin perfusion pressure (SPP) is used to evaluate the tissue blood pressure, and SPP >40 mmHg is thought to be necessary to promote wound healing.^5)^ To achieve this level, the angiosome concept, which was first reported by Taylor and Palmer et al.^6)^ has been applied to CLTI treatment. The essence of the angiosome concept is that direct revascularisation (DR) of angiosomes provides direct arterial flow to ischemic tissue and offers a better chance of wound healing. However, the effects of DR on distal bypass surgery and endovascular treatment (EVT) are controversial. The importance of DR has been established in the EVT field.^7^^–^^9)^ During bypass surgery, DR may be preferable, but it is not always possible and, instead, IR is often performed. Some vascular surgeons believe distal bypass to any of three arteries improves the blood supply beyond any given angiosome.^10)^ In this study, repeated SPP measurements were obtained to evaluate whether revascularisation with distal bypass improves tissue blood pressure beyond a focal angiosome.

## Patients and Methods

This was a prospective multicentre observational study, and clinical data of patients who underwent distal bypass surgery at four hospitals (Ishinomaki Red Cross Hospital, Japan Community Health Care Organization Sendai Hospital, Iwate Prefectural Isawa Hospital, Iwate Prefectural Chubu Hospital) were collected and analysed. The ethics committee approved the study (approval no. 19–17), and formal written informed consent was obtained from each patient. Background demographic data and clinical outcomes, including sex, age, hypertension, dyslipidaemia, diabetes mellitus, insulin use, history of ischemic heart disease, cerebrovascular disease, chronic obstructive pulmonary disease, and regular haemodialysis were obtained from medical records.

### Study protocol

Enrolment was limited to patients who were diagnosed with CLTI (Rutherford classification 5) and who underwent distal bypass surgery between September 2019 and May 2021. We choose distal bypass when both the anterior tibial artery (ATA) and posterior tibial artery (PTA) are obstructed, and distal bypass was defined as surgery in which the distal anastomosis below the popliteal artery and the proximal anastomosis site were not considered. SPPs were measured repeatedly at the dorsal and planta aspects of the foot, at the midpoint of the third metatarsal bone, both preoperative and postoperatively. The first SPP measurement after surgery was performed within 7 days, and SPP measurements were repeated once or twice per week until postoperative day 28. The measurement site was marked with an oil-based pen to avoid a measurement site shift. If the patient was discharged before postoperative day 28, the SPP measurements were continued in the outpatient department if possible. All measurements were performed using the PAD 3000 (Kaneka Medical Products, Osaka, Japan). Preoperative and postoperative ankle-brachial pressure (ABI) was also measured.

### Exclusion criteria

Patients who did not have normal judgement, and those who had extensive tissue loss were excluded. Patients who underwent trans-metatarsal or more proximal amputation or bypass occlusion before postoperative day 28 were also excluded.

### Definition of revascularisation area and wound location

According to the angiosome concept, the dorsal pedis was defined as the direct revascularized angiosome (DRA) if the distal anastomosis site was ATA or the dorsal pedal artery regardless of the wound location. Similarly, the plantar pedis was defined as the DRA if the distal anastomosis site was PTA or the plantaris artery. The rest of angiosomes were defined as the indirect revascularized angiosome (IRA). If the wound was located on a border between angiosomes or multiple wounds were located among different angiosomes, the angiosome mostly affected was recorded. According to a previous report,^11)^ if revascularisation of the digital ulcer was achieved via ATA including the dorsal pedal artery or PTA including the planter artery, we defined this as direct revascularized regardless of the side on which the ulcer was located. The present study included wounds located in the following areas. Digital: 19 wounds, dorsal pedis: 4 wounds, planta pedis: 4 wounds, heel: 2 wounds. Based on the previously defined criteria, 24 wounds underwent direct revascularization, while 5 wounds were revascularized indirectly.

### Definition of the wound healing

Wound healing was judged by a skilled vascular surgeon and defined as complete epithelialisation.

### Surgical procedures

Proximal and distal anastomosis sites were determined by each operator based on preoperative CT angiography, angiography, and ultrasound imaging. A bypass conduit was employed to single reverse the great saphenous vein. An anastomosis was created with polypropylene surgical sutures. Intraoperative angiography was performed if necessary.

### Statistical methods

Continuous and discrete variables were examined using the Mann–Whitney U test and Fisher’s exact test, respectively. The wound healing rate was analysed using a cumulative incidence curve and Gray’s test. Analyses were performed using Microsoft Excel 2016 (Microsoft Inc., Redmond, WA, USA) and EZR ver. 3.3.1 (https://www.jichi.ac.jp/), and *p*-values < 0.05 were considered significant.

## Results

### Patient characteristics

Based on predefined criteria, 29 limbs in 27 patients (22 males and five females, average age: 70.2 ± 9.3 years) were enrolled. The follow-up period was 281 (IQR = 174–607) days. During the follow-up period, nine patients died and no bypass occlusion was observed. All limbs were Rutherford 5. There were 19 registered wounds on the digital, four on the ATA, four on the PTA, and two on the peroneal artery angiosome. In total, 486 SPPs were collected from 58 SPP measurement sites. A detailed overview of patient characteristics and surgical information is shown in **Table 1**.

**Table:** Table 1 Patient characteristics and surgery

Variable	No. (%) or mean ± SD	
Sex	*n* = 27	
Male	22	81%
Female	5	19%
Age		
All	70.2 ± 9.3	
Medical history	*n* = 27	
Hypertension	25	93%
Dyslipidaemia	17	63%
Diabetes mellitus	19	70%
Insulin use	10	37%
Ischaemic heart disease	13	48%
Cerebrovascular disease	5	19%
Chronic obstructive pulmonary disease	9	33%
Regular haemodialysis	21	78%
Smoking history	21	78%
Rutherford classification	*n* = 29	
Rutherford 5	29	100%
Wound location	*n* = 29	
Digital	19	66%
Anterior tibial artery angiosome	4	14%
Posterior tibial artery angiosome	4	14%
Peroneal artery angiosome	2	7%
Surgery		
Surgical time (median)	291 (245–430)	min
Blood loss (median)	152 (66–377)	mL
Inflow repair	6	21%
Bypass conduit	*n* = 29	
Single reversed great saphenous vein	29	100%
Proximal anastomosis	*n* = 29	
Common femoral artery	8	28%
Superficial femoral artery	3	10%
Above-the-knee popliteal artery	4	14%
Below-the-knee popliteal artery	14	48%
Distal anastomosis	*n* = 29	
Anterior tibial artery	15	52%
Dorsal artery	7	24%
Posterior tibial artery	6	21%
Plantar artery	1	3%

SD: standard deviation

### Bypass surgery and amputation

The distal bypass surgery time was 291 min (IQR = 245–430 min) and blood loss was 152 mL (IQR = 66–377 mL). Six patients received inflow repair, including femoro-popoliteal artery bypass, iliac/superficial femoral artery (SFA) stenting, SFA angioplasty, and thromboendoarterectomy. The proximal anastomosis sites were the common femoral artery (n = 8), SFA (n = 3), the above-the-knee popliteal artery (n = 4), and the below-the-knee popliteal artery (n = 14). The distal anastomosis sites were the ATA (n = 15), the dorsal artery (n = 7), PTA (n = 6), and the planter artery (n = 1). Single reversed great saphenous vein grafts were used in all patients. All patients received debridement or minor amputation.

### Ankle brachial pressure

Pre-ABI and post-ABI were 0.56 ± 0.24 and 0.98 ± 0.20 (p = 0.01).

### SPP transition of the direct and indirect revascularized areas

**Figure 1:**
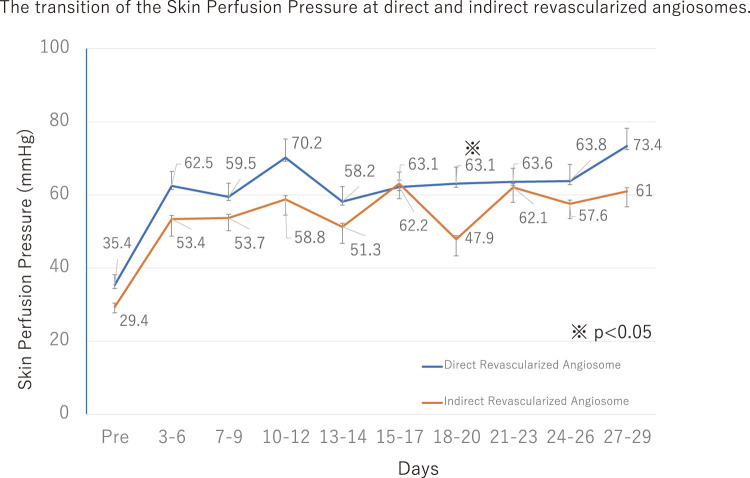
Fig. 1 SPP of the direct and IRA. SPP reached > 40 mmHg immediately in the DRA and IRA with a similar time course. Although SPP at the DRA seemed to be superior compared to the IRA, no significant differences were observed between SPP at the direct and indirect revascularized areas except on days 18–20 (p = 0.02). SPP: skin perfusion pressure; IRAs: indirect revascularized angiosomes; DRA: direct revascularized angiosome

The SPP transition at the DRA and IRA is shown in **Fig. 1**. A significant difference was observed between revascularized areas after 18–20 days (p = 0.03).

### Grouping

**Figure 2:**
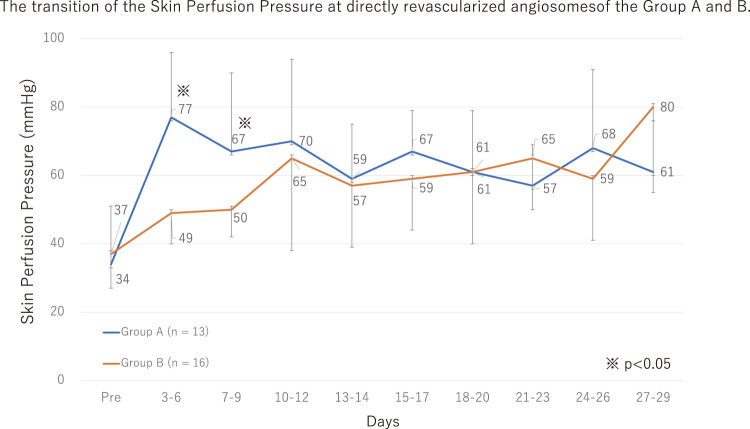
Fig. 2 The groups were divided into groups A (n = 13, 45%; SPP peak days occur within 9 days) and group B (n = 16, 55%; peak days occur after 10 days). The SPP transition in each group is shown. Between postoperative 3–9 days, group A had significantly higher SPPs than group B, but the significant differences disappeared after postoperative 10 days. SPP: skin perfusion pressure

The patients were divided into two groups based on the number of postoperative days for the SPP peak at the DRA. The SPP peak was defined as either the actual peak of the SPP or the first day when it reached an actual peak of 20%. The groups were divided into group A (n = 13, 45%; SPP peak days occur within 9 days) and group B (n = 16, 55%; peak days occur after 10 days). The SPP transition in each group is shown in **Fig. 2**. Between postoperative 3 and 9 days, group A had significantly higher SPPs than group B, but the significant differences disappeared after postoperative 10 days.

### Wound healing and DR/IR

Gangrene was observed in 29 limbs, including 24 in which the wounds were direct revascularized and five in which they were indirect revascularized. The wounds of 20 of the 29 limbs healed and the wound healing time was 81 days (IQR = 39–149). Four patients died before wound healing and two patients underwent a major amputation before wound healing. Gangrene still occurred in three limbs. Wounds on 15 (63%) of 24 DR limbs and five (100%) of five IR limbs eventually healed.

**Figure 3:**
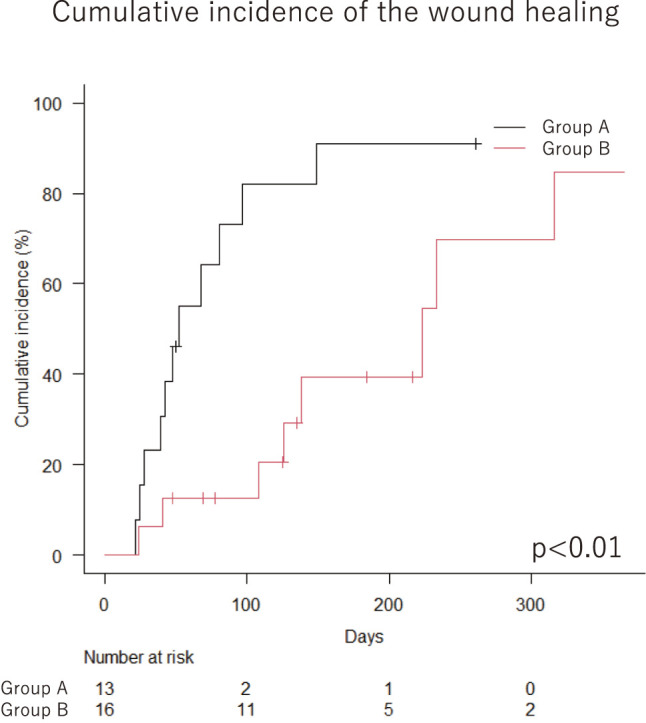
Fig. 3 Cumulative incidence curve of wound healing in each group. The wound healing rate in group A was significantly higher (p = 0.01) and wound healing time was significantly shorter (p = 0.04) than those in group B. Details of each group are also shown in **Table 2**. Figures are adapted from Distal Bypass Improves Skin Perfusion Pressure at the Entire Foot Regardless of Angiosomes in Patients with Chronic Limb-Threatening Ischaemia, presented at Vascular Annual Meeting 2023 in National Harbor, MD, June 14–17, F. SERIZAWA, with kind permission from ELSEVIER (License Number: 5673890665268)

Among the 13 wounds grouped in A (SPP peak was within 9 days after surgery), wound healing was observed in 12 (92%), and the healing time was 52 days (IQR = 39–97). The wound healing rate in group B (SPP peak was after 10 days) was 40.0% (8 of 16 limbs), and the healing time was 175 days (IQR = 90–253) shown in **Table 2** and **Fig. 3**.

**Table:** Table 2 Clinical data of each group

	Group A (n = 13)	Group B (n = 16)	p-Value
Wound healing	12 (92%)	8 (50%)	0.01
Healing time Median days (IQR)	52 (39–97)	175 (90–253)	0.02
Died or amputation before heal	1 (8%)	5 (31%)	0.18
Hypertension	11 (84%)	12 (80%)	0.60
Dyslipidaemia	7 (54%)	8 (53%)	1.00
Diabetes mellitus	7 (54%)	11 (69%)	0.45
Insulin	2 (15%)	7 (44%)	0.22
Ischaemic heart disease	4 (31%)	8 (50%)	0.44
Cerebrovascular disease	2 (15%)	2 (13%)	1.00
Chronic obstructive pulmonary disease	3 (23%)	4 (25%)	1.00
Haemodialysis	9 (69%)	12 (75%)	1.00
Smoking history	10 (77%)	11 (69%)	0.66

IQR: interquartile range

## Discussion

According to the angiosome concept, the foot is divided into three anatomical territories and each source artery corresponds to skin and deep tissue perfusion. SPP >40 mmHg is thought to be necessary to promote wound healing.^5)^ The angiosome concept has been applied to CLTI treatment, and DR has been emphasized.^12)^ However, the effects of angiosomes and DR during bypass surgery and EVT are not well understood and may not be equal. While the superiority of the angiosome concept and DR seem to be accepted in EVT, ^7^^–^^9)^ their usefulness remains controversial in bypass surgery.^10^^,^^13^^,^^14)^ However, some vascular surgeons believe that bypass surgery increases blood flow to the DR and IR areas, but the evidence is insufficient to fully support this theory. The question remains whether bypass can supply enough blood to the wound beyond the angiosome.

### DRA versus IRA

In the present study, we measured SPPs at 10 time points before and after surgery and discovered that the SPPs increased in both DRA and IRA with a similar time course (**Fig. 1**). In addition, SPP >40 mmHg, which is necessary to promote wound healing, was achieved in both angiosomes at all time points. These data indicate that distal bypass surgery increased blood pressure enough to heal the wounds in the whole foot, regardless of the revascularized angiosomes, and this effect persisted for at least 1 month. Successful bypass usually increases arterial pressure effectively and restores blood flow and collateral blood from beyond the areas of the angiosomes.^15)^ While primary patency of distal bypass at 1 year is 61%–79%,^16^^–^^18)^ the restenosis and occlusion rate of angioplasty for infrapopliteal lesions at 3 months is 73%.^19)^ These results indicate that elevated blood pressure with EVT decreases earlier than that after bypass and results in decreased blood flow in the tissue. Regardless of the revascularisation method, it may be difficult to sustain sufficient tissue blood pressure beyond a given angiosome with low blood flow, suggesting why the angiosome concept is not as important for wound healing as previously reported^8^^,^^10^^,^^13)^ and why wound healing after bypass may be better than EVT.^9)^ However, the pedal arch and side collaterals strongly affect wound healing,^8)^ although we did not evaluate the quality of the pedal arch in this study. As this was a multicentre study, the radiation equipment in each hospital was different and the quality of the available angiography imaging was insufficient in some cases. According to a previous report, only 16% of CLTI patients have a patent pedal arch^20)^ and most of the patients enrolled in the present study may not have had a patent pedal arch, so further study is needed. In the present study, six patients received inflow repair. Inflow repair has the potential to impact both direct and indirect angiosomes. However, given that our study includes cases where both the ATA and PTA are occluded, even with inflow repair, blood flow to the foot and ankle is believed to be supplied either through collateral pathways or via the peroneal artery. Consequently, we anticipate that the influence on blood flow to the direct or indirect angiosomes, as investigated in this study, is limited.

### Wound healing

In our study, the enrolled patients had only small areas of gangrene and 18 of the 29 wounds were digital ulcers that received blood supply from two angiosomes. According to predefined criteria, 24 of the 29 (83%) wounds were direct revascularized and 5 of the 29 were indirect revascularized; 63% of the direct revascularized wound and 100.0% of the indirect revascularized wound healed. These data suggest that the angiosome concept may not be important for wound healing but it is difficult to make well-grounded conclusions based on our data due to the limited number of patients and the data bias. However, we focused on the timing of the SPP peak. The wound healing rate in group A was significantly higher (p = 0.01) and wound healing time was significantly shorter (p = 0.04) than in group B. An early SPP peak in our data indicated a better clinical outcome and a late SPP peak or unimproved SPP cases resulted in high mortality before wound healing. Both Group A and Group B ultimately met the SPP (>40 mmHg) requirement necessary for wound healing. However, the reasons for variations in the wound healing rates based on the timing of SPP elevation were not conclusively identified in this study. It is well-known that with the progression of occlusive arterial disease, microvascular deterioration occurs, leading to a condition known as desert foot. The differences in the timing of SPP elevation may possibly reflect the extent of microvascular deterioration in conditions such as desert foot. We hypothesized that the peak timing of SPP indicates the quality of the arterial-arterial connection and arterial plexus, but further study is needed.

### Limitations

Some limitations of this study should be noted. First, this was a non-randomized analysis. Second, it was impossible to preclude all known or unknown factors influencing the SPP measurements and our dataset may include measurement errors. Third, the quality of the pedal arch was not evaluated. Fourth, the SPP data were collected from patients who underwent distal bypass, and EVT cases were not included.

## Conclusion

Distal bypass improves SPP >40 mmHg in both direct and IRAs of CLTI patients. These data indicated that distal bypass improves tissue blood flow at the entire foot regardless of angiosomes and early SPP peak timing could be an indicator of wound healing.

## Disclosure Statement

All authors have no conflict of interest.

## Author Contributions

Study conception: FS, YN, and MH

Data collection: FS, YN, MH, YT, HS, MO, and KK

Analysis: FS and MH

Investigation: FS

Manuscript preparation: FS, MH, and DA

Funding acquisition: None

Critical review and revision: All authors

Final approval of the article: All authors

Accountability for all aspects of the work: All authors.
